# Characterization of lncRNA *LINC00520* and functional polymorphisms associated with breast cancer susceptibility in Chinese Han population

**DOI:** 10.1002/cam4.2893

**Published:** 2020-01-29

**Authors:** Qiaoyun Guo, Linping Xu, Rui Peng, Yan Ma, Yanli Wang, Feifei Chong, Mengmeng Song, Liping Dai, Chunhua Song

**Affiliations:** ^1^ Department for Endemic Disease Control and Prevention Henan Provincial Center for Disease Control and Prevention Zhengzhou Henan PR China; ^2^ Department of Epidemiology and Statistics College of Public Health Zhengzhou University Zhengzhou Henan PR China; ^3^ Henan Key Laboratory of Tumor Epidemiology Zhengzhou Henan PR China; ^4^ Department of Teaching and Research Henan Tumor Hospital Zhengzhou Henan PR China; ^5^ Department of Teaching and Research Third Affiliated Hospital of Zhengzhou University Zhengzhou Henan PR China; ^6^ Henan Luoyang Orthopedic Hospital (Henan Provincial Orthopedic Hospital) Zhengzhou China; ^7^ Henan Provincial Orthopedic Institute Zhengzhou China

**Keywords:** breast cancer, *LINC00520*, lncRNA, single‐nucleotide polymorphisms, susceptibility

## Abstract

**Background:**

The aim was to evaluate the association between the *LINC00520* genetic polymorphisms and breast cancer (BC) susceptibility.

**Methods:**

Nine single‐nucleotide polymorphisms (SNPs) on *LINC00520* genotyping were performed in 504 BC patients and 505 cancer‐free controls in Chinese Han population to study the relationship between *LINC00520* polymorphism and BC susceptibility. qRT‐PCR and luciferase tests were used to explore how rs12880540 affected the expression of *LINC00520*.

**Results:**

The genotype GG (OR:3.58, 95%CI:1.32‐9.69) in rs8012083 increased the risk of triple‐negative BC. The genotype GG (OR:0.31, 95%CI:0.14‐0.69) in rs8012083, the genotype AA (OR:2.74, 95%CI:1.01‐7.42) in rs2152275, and genotype TG (OR:1.62, 95%CI:1.04‐2.52) in rs12880540 were associated with HER‐2 status. The dominant (OR:0.65, 95%CI:0.45‐0.95) and overdominant genetic model (OR:0.67, 95%CI:0.46‐0.98) consistently showed that rs11622641 T was significantly associated with lower risk of BC. Similarly, the recessive genetic model (OR:1.57, 95%CI:1.07‐2.30) of rs12880540 and the dominant (OR:1.62, 95%CI:1.24‐2.11) and overdominant (OR:1.56, 95%CI:1.19‐2.03) genetic model of rs2152278 may increase the risk of BC. The relative expression of *LINC00520* increased linearly with the increase in the number of rs12880540 mutations. rs12880540 alleles were due to the interaction between *LINC00520* and miR‐3122 at T, but the mutation of rs12880540 G > T had no effect on the binding ability of *LINC00520* and miR‐3122.

**Conclusion:**

A genetic variant of rs8012083 in *LINC00520* may be used as a biomarker for triple‐negative BC after further evaluation of diagnostic tests. The genetic variant of *LINC00520* was related to the susceptibility of BC, and rs12880540 might affect the corresponding mRNA expression of lncRNA *LINC00520*.

## Graphical abstract

In our epidemiology study, nine single‐nucleotide polymorphisms (SNPs) in lncRNA *LINC00520* were selected for genotyping among 504 breast cancer cases and 505 healthy controls. The 504 cases are all new breast cancer patients which were pathologically confirmed and the controls were from an epidemiological survey of cardiovascular disease in Henan Province with 20 000 participants and they were included without a history of cancer or other diseases. A genetic variant of rs8012083 in *LINC00520* could be used as a biomarker for triple‐negative BC. The genetic variants of lncRNA *LINC00520* were associated with the susceptibility of BC, and rs12880540 might affect the corresponding mRNA expression of lncRNA *LINC00520*.

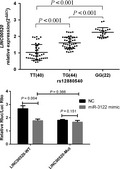

## 1 INTRODUCTION

Breast cancer (BC) is the second most malignant tumor in the world, ranked after lung cancer. BC remains the leading cause of cancer deaths in Chinese women, placing a huge burden on public health.^1^ In China, new BC cases accounted for 12.2% of all new cancer cases and 9.6% of all deaths.^2^ With the Chinese population increasing and accelerated aging, the number of new BC cases has been continually rising in recent years and the age of onset tended to be younger.^3^ The high incidence and mortality of BC in China have become a public problem that seriously threatened women's physical and mental health.^4^ Finding the specific susceptibility biomarker and identifying individuals with higher risk of BC could be helpful to improve the early diagnostic rate of BC.

With the development of high‐throughput technology, emerging evidence demonstrated that genetic alterations, including single‐nucleotide polymorphisms (SNPs), insertions, deletions, and copy number variations, were associated with the risk of various diseases including BC.^5^, ^6^ Notably, SNPs, especially in irregular regions of protein‐coding and related with diseases, have been extensively studied and reported widely.^7^ The expression of the same lncRNA transcripts varies by different health conditions, age, tissues, even cells, which can indicate their potential as possible biomarkers and be predictive of diagnosis and prognosis of diseases.^8^, ^9^ Enormous studies have proven that lncRNAs were associated with tumorgenesis, progression, metastasis, and drug resistance, functioning as oncogenes, tumor suppressor genes, or both, involving in shaping multiple biological characteristics of cancer at transcriptional, posttranscriptional, and epigenetic levels.^10^, ^11^, ^12^ In addition, the abnormal expression levels of different lncRNAs with oncogenic and tumor suppressor functions have been frequently found in the development of BC.^13^ Particularly, adverse rules of miRNAs‐sponging lncRNAs, involved in ceRNA networks (ceRNETs), have been shown in BC.^14^ Eades discovered lincRNA‐ROR/miR‐145/ARF6 mRNA networks interacting with metastasis and prognosis in triple‐negative BC in 2015.^15^

Long intergenic nonprotein coding RNA 520 (*LINC00520*) is located at human chromosome 14q22.3. *LINC00520*, a highly conserved long‐chain noncoding RNA with a length of about 20kb, is widely expressed in various tissues. A study reported that *LINC00520* was overexpressed in laryngeal squamous cell carcinoma tissue.^16^ The upregulation of *LINC00520* expression might play an important biological role in the metastasis of laryngeal squamous cell carcinoma. The expression of *LINC00520* was upregulated in primary nasopharyngeal carcinoma.^17^ In addition, the level of *LINC00520* was significantly increased in renal cell carcinoma.^18^ *LINC00520* promoted the migration of BC cells induced by *Src*, *STAT3*, and *PI3K* and played a functional role in BC.^19^ Although it was reported that the lncRNA *LINC00520* was associated with BC, the complexity of its function was also determined by the complexity of the lncRNAs structure. However, the association among *LINC00520* SNPs and the susceptibility of BC and its mechanism is not clear yet. In this study, the combined database, literature retrieval, and bioinformatics techniques were used to screen lncRNA and its SNP site associated with BC. The association between long noncoding RNA genetic variation and BC susceptibility was studied, as well as biological function was explored by the method of molecular epidemiology.

## 2 MATERIALS AND METHODS

### 2.1 Study subjects

A total of 1009 subjects were enrolled in the study, including 504 cases with BC and 505 age‐matched (±2 years) healthy controls (Table 1). Subjects were included in accordance with strict standards: (a) Patients with BC must have a definite pathological diagnosis of new cases; (b) The patients with BC were selected from the First Affiliated Hospital and the Third Affiliated Hospital of Zhengzhou University from 2013 to 2015; (c) Patients with BC have never received radiotherapy or chemotherapy or any other treatment before; (d) Except BC, there were no other diseases in all cases.

**Table 1:** Basic characteristics of 504 breast cancer cases and 505 healthy controls

Variables	Cases (%)	Controls (%)	*P* a	OR (95%CI)
	n = 504	n = 505		
Age (mean ± SD)	48.00 ± 9.85	48.15 ± 9.61	.906^b^	
Age at menarche (mean ± SD)	14.21 ± 1.70	13.97 ± 1.75	*.030* b	
Menopause state				
Premenopause	320 (63.5)	294 (58.2)		1.000
Postmenopause	184 (36.5)	211 (41.8)	.086	0.801 (0.622‐1.032)
Age at menopause				
≤50	129 (70.1)	146 (69.2)		1.000
>50	55 (29.9)	65 (30.8)	.844	0.958 (0.623‐1.472)
Number of pregnancies				
≤1	53 (10.5)	94 (18.6)		1.000
2	129 (25.6)	177 (35.0)	.215	1.293 (0.861‐1.940)
3	135 (26.8)	128 (25.3)	.262	1.898 (0.620‐5.816)
≥4	187 (37.1)	106 (21.0)	*.043*	*3.175 (1.037‐9.722)*
Number of abortion				
≤1	340 (67.5)	345 (68.3)		1.000
2	105 (20.8)	117 (23.2)	.545	0.911 (0.673‐1.233)
3	35 (6.9)	30 (5.9)	.735	0.913 (0.537‐1.551)
≥4	24 (4.8)	13 (2.6)	.308	1.444 (0.713‐2.926)
Breastfeeding				
No	121 (24.0)	94 (18.6)		1.000
Yes	383 (76.0)	411 (81.4)	*.037*	*0.724 (0.535‐0.980)*
Family history				
No	461 (91.5)	481 (95.2)		1.000
Yes	43 (8.5)	24 (4.8)	*.017*	*1.869 (1.116‐3.130)*
ER				
Negative	149 (30.3)			
Positive	342 (69.7)			
PR				
Negative	191 (39.1)			
Positive	298 (60.9)			
HER‐2				
Negative	136 (29.3)			
Positive	328 (70.7)			

Abbreviations: ER, estrogen receptor; HER‐2, human epidermal growth factor receptor‐2; PR, progesterone receptor.

a Two‐sided Chi‐square test, *P* < .05 was statistically different.

b Student's *t* test.

The italic values represent *P* < .05.

The control was collected from the site of an epidemiological survey of cardiovascular disease involving more than 20 000 people in Henan Province during the same time. The healthy controls were matched with the BC group according to the age (±2 years), who were healthy without any history of cancer or any other chronic disease. None of the participants had any affinity.

All subjects volunteered to participate in the study and signed informed consent. The study has been approved by the Medical and Health Research Ethics Committee of Zhengzhou University.

### 2.2 Data collection

The data of the patients with BC were obtained through medical records of the hospital, including names, ages, nationality, place of origin, menarche age, menopause status, whether there was a history of breastfeeding and childbearing, cancer history of first‐degree relatives (parents, siblings, and children), second‐degree relatives (grandparents, uncles, aunts), along with estrogen receptor (ER), progesterone receptor (PR), and human epidermal growth factor receptor‐2 (Her‐2).

The data of controls were collected by face‐to‐face questionnaire designed by experts, asked by trained investigators. The content of the data was the same as BC cases. All the collected information was entered parallel with EpiData software. The whole process of investigation was carried out under strict quality control, including collection, collation, and entry of information.

### 2.3 DNA extraction

Peripheral blood (5 mL) was collected from each subject using an anticoagulation vessel containing ethylenediaminetetraacetic acid (EDTA) for DNA extraction and genotyping. The genomic DNA was extracted using genomic DNA extraction kit (DK601‐02 centrifugal column type) of Shanghai Lifefeng Biotech Co., Ltd and the operation process was carried out in accordance with the instructions strictly. All the extracted genomic DNA was labeled, measured, and finally stored in −80°C refrigerator for future use.

### 2.4 SNPs selection and genotyping

LncRNA database (http://www.lncrnadb.org/), NCBI database (https://www.ncbi.nlm.nih.gov/gene/), Ensembl database (http://asia.ensembl.org/Homo_sapiens/Info/Index), and 1000 Genomes Browser (http://browser.1000genomes.org/index.html) were used to determine the location of *LINC00520*. We selected the tagger SNPs in region of *LINC00520* by lncRNASNP2 database (http://bioinfo.life.hust.edu.cn/lncRNASNP) based on Chinese Han population data of HapMap Project (HapMap Rel28, NCBI B36) with the criteria of a minor allele frequency (MAF) higher than 0.1 in Chinese Han population and the pairwise correlation was set as *r* ^2^ less than .8, which can reflect the degree of chain reaction between SNP. Secondly, we screened the SNP located in the Promoter Flanking Region and Enhancer regions of *LINC00520*. Then, lncRNASNP2 (http://bioinfo.life.hust.edu.cn/lncRNASNP) database was used to predict the potential miRNA binding sites of the selected SNPs. RNAfold website (http://rna.tbi.univie.ac.at/cgibin/RNAWebSuite/RNAfold.cgi) was utilized to predict the influence of the selected SNPs in *LINC00520* on the secondary structure of *LINC00520* (Table S1). Finally, nine SNPs (rs11622641, rs7157819, rs12880540, rs2152275, rs4144657, rs2152278, rs7142488, rs8008130, and rs8012083) in *LINC00520* were selected for further research (Table S2).

In this study, rs11622641, rs7157819, rs12880540, and rs2152275 located in *LINC00520* were genotyped by SNPscan. Because of the low score of other SNPs, SNPscan method was not suitable. Polymerase chain reaction‐restriction fragment length polymorphism (PCR‐RFLP) technique was used for genotyping of rs4144657, rs2152278, and rs7142488 in *LINC00520*, while rs8008130 and rs8012083 using created restriction site PCR (CRS‐RFLP). While there was no suitable restriction endonuclease near the mutation site, we needed to change a base on the primer so that a new restriction site could be formed with the base of the mutation site, thus CRS‐RFLP method was used. Primers for PCR amplification were designed using primer 6.0 software. NCBI BLAST website (https://www.ncbi.nlm.nih.gov/tools/primer-blast/) was used to evaluate the specificity of primers. Gradient PCR technique was used to standardize the conditions of DNA amplification and optimize the annealing temperature for the primers set. The suitable restriction enzymes were selected by WatCut website (http://watcut.uwaterloo.ca/template.php; Table S3). Moreover, approximately 5% of samples were randomly selected for Sanger sequencing, yielding a 100% concordance rate between the two methods.

### 2.5 Quantitative real‐time PCR analysis

The relative expression of *LINC00520* was detected by the Eco Real‐Time PCR System (Illumina). The primers of *LINC00520* and *GAPDH* were synthesized by TaKaRa Biotechnology Co. Ltd. The reaction conditions of qRT‐PCR were performed in strict accordance with the following instructions. All specific primers used in quantitative RT‐PCR were shown below, *LINC00520* primer: 5′‐AAGCAGGACACAATTACAAC‐3′ and 5′‐GCAGGTCCGAGGTATTCGTC‐3′, *GAPDH* primer: 5′‐CGGAGTCAACGGATTTGGTCGTAT‐3′ and 5′‐AGCCTTCTTCATGGTGGTGAAGAC‐3′. All samples were independently measured in triplicate. Finally, the relative expression of the gene was the average of three replicates per sample. The relative expression of *LINC00520* was calculated by 2^−ΔΔCT^ method, and *GAPDH* was used as the internal reference gene (X ± SD).^20^

### 2.6 Cell culture, transfection, and luciferase reporter assay

To further examine whether rs12880540 could have impact on the binding of miR‐3122 and *LINC00520*, predicted by lncRNASNP2 database (Figure S1), 293T cell lines were obtained and grown in DMEM supplemented with 10% fetal bovine serum (GIBCO) in a humidified atmosphere of 5% CO2 at 37°C. All cell lines were never subcultured for more than 3 months and sequenced by DNA using the Applied Biosystems Amp F/STR Identifier kit and last performed in September 2018. The miR‐3122 mimics and negative control (NC) were synthesized in Genecreate. For transfection assays, 293T cells were seeded in 48‐well plates and simultaneously transfected with PGL3‐basic‐luc vector and miR‐3122 or NC mimics using Lipofectamine 3000 (Invitrogen). After 48 hours, cells were harvested, and Renilla luciferase/Firefly luciferase activities between different alleles were detected and analyzed according to the manufacturer's instruction (dual luciferase assay system, Promega). All experiments were performed independently in triplicate.

### 2.7 Quality control

Negative control was performed for each batch of PCR amplification to avoid false positive of sample contamination. About 5% of samples were randomly selected for sequencing to ensure the reliability of genotyping results. Figure S2 showed the PCR‐RFLP and CRS‐RFLP techniques, while the sequencing results were shown in Figure S3. The qRT‐PCR experiments were repeated three times independently to reduce the measurement error.

### 2.8 Statistical analysis

The sample size of the study (n = 460) was calculated using Power Analysis and Sample Size (PASS) software based on study power (0.9) and minimal alleles frequency (0.1). *Student's* *t* test was used for continuous normal distribution variables and Chi‐square test was used to classify variables. Hardy‐Weinberg equilibrium (HWE) was examined to compare the observed genotype frequencies of cancer‐free controls with the expected genotype frequencies by goodness‐of‐fit Chi‐square test. Odds ratios (ORs) and its 95% confidence intervals (CIs) were calculated to evaluate the association between SNPs and BC susceptibility adjusted for potential confounding factors, including age, age of menarche, age of menopause, menopausal status, pregnancy number, abortion number, breastfeeding history, and family history of BC in first‐degree relatives. MDR 2.0 software was used to analyze interaction between polymorphic sites in lncRNA gene and reproductive factors. SHEsis (http://analysis.bio-x.cn/myAnalysis.php) was conducted to calculate the difference of haplotype frequencies in both patients and controls. The input of data was carried out by EpiData software with double parallel entry. The statistical analysis was performed by SPSS 23.0 software.

## 3 RESULTS

### 3.1 Characteristics of the participants

The average age of BC cases was 48.00 years (±9.85), while that of the control group was 48.15 years (±9.61). No statistical differences were found in the distribution of age, menopause state, age at menopause, and number of abortion between the BC cases and cancer‐free controls (all *P* > .05). The mean age at menarche was 14.21 years (±1.70) in BC patients and 13.97 years (±1.75) in controls (*P* = .03). Breastfeeding was a protective factor (OR:0.73, 95%CI:0.54‐0.98), while carrying family history of tumor was a risk factor for BC cases (OR:1.87, 95%CI:1.12‐3.13). Approximately, half of the patients were positive in ER, PR, and Her‐2 (342 [69.7%], 298 [60.9%], and 328 [70.7%], respectively).

### 3.2 The associations between SNP genotypes and risk of BC

As indicated in Table 2, the genotype distributions of the nine SNPs in control group all conformed to HWE (*P* > .05). The relationship between SNPs and BC risk based on different genetic models is summarized in Table 2. When after adjusting the confounding factors in logistic regression analysis, the unconditional multiple logistic regression based on the dominant (OR:0.65, 95%CI:0.45‐0.95) and overdominant genetic model (OR:0.67, 95%CI:0.46‐0.98) consistently showed that rs11622641 T was significantly associated with lower risk of BC. Similarly, the recessive genetic model (OR:1.57, 95%CI:1.07‐2.30) also showed that the individuals with 12880540 G had increased risk of BC. The dominant (OR:1.62, 95%CI:1.24‐2.11) and overdominant (OR:1.56, 95%CI:1.19‐2.03) genetic model also revealed that rs2152278 T could increase the risk of BC.

**Table 2:** Association between nine SNPs of *LINC00520* and breast cancer susceptibility

SNPs genetic model	Genotype	Cases (%)	Controls (%)	*P* a	*P* b	OR (95%CI)	*P* c
		n = 504	n = 505				
rs11622641				*.030*	.995		
Codominant	CC	445 (88.3)	417 (82.6)	Reference		1.000	
	CT	57 (11.3)	84 (16.6)	*.014*		*0.666 (0.453‐0.979)*	*.039*
	TT	2 (0.4)	4 (0.8)	.439		0.359 (0.056‐2.289)	.278
Dominant	CT + TT/CC					*0.650 (0.445‐0.949)*	*.026*
Recessive	TT/CC + CT					0.375 (0.059‐2.393)	.299
Overdominant	CT/CC + TT					*0.670 (0.455‐0.984)*	*.041*
rs7157819				.556	.821		
Codominant	CC	375 (74.4)	391 (77.4)	Reference		1.000	
	CT	119 (23.6)	105 (20.8)	.288		1.054 (0.768‐1.445)	.746
	TT	10 (2.0)	9 (1.8)	.819		0.971 (0.372‐1.445)	.952
Dominant	CT + TT/CC					1.047 (0.770‐1.423)	.770
Recessive	TT/CC + CT					0.959 (0.369‐2.492)	.931
Overdominant	CT/CC + TT					1.054 (0.769‐1.445)	.742
rs12880540				.069	.460		
Codominant	TT	208 (41.3)	218 (43.2)	Reference		1.000	
	TG	222 (44.0)	237 (46.9)	.893		1.025 (0.776‐1.354)	.863
	GG	74 (14.7)	50 (9.9)	*.041*		*1.551 (1.034‐2.328)*	*.034*
Dominant	TG + GG/TT					1.098 (0.843‐1.429)	.488
Recessive	GG/ TG + TT					*1.566 (1.069‐2.295)*	*.021*
Overdominant	TG/GG + TT					0.948 (0.730‐1.232)	.690
rs2152275				.886	.680		
Codominant	TT	271 (53.8)	265 (52.5)	Reference		1.000	
	TA	199 (39.5)	207 (41.0)	.646		1.001 (0.763‐1.315)	.992
	AA	34 (6.7)	33 (6.5)	.997		1.128 (0.658‐1.933)	.661
Dominant	TA + AA/TT					1.018 (0.784‐1.322)	.892
Recessive	AA/TA + TT					1.127 (0.667‐1.906)	.655
Overdominant	TA/AA + TT					0.988 (0.758‐1.289)	.930
rs8008130				.702	.644		
Codominant	CC	418 (82.9)	427 (84.6)	Reference		1.000	
	CA	79 (15.7)	73 (14.5)	.598		1.165 (0.810‐1.674)	.411
	AA	7 (1.4)	5 (1.0)	.575		1.448 (0.410‐5.111)	.565
Dominant	CA + AA/CC					1.182 (0.831‐1.681)	.352
Recessive	AA/CA + CC					1.418 (0.403‐4.997)	.586
Overdominant	CA/AA + CC					0.862 (0.600‐1.239)	.424
rs4144657				.252	.122		
Codominant	CC	147 (29.2)	126 (25.0)	Reference		1.000	
	CT	267 (53.0)	275 (54.5)	.235		0.859 (0.632‐1.168)	.333
	TT	90 (17.9)	104 (20.6)	.133		0.756 (0.511‐1.117)	.160
Dominant	CT + TT/CC					1.204 (0.897‐1.615)	.216
Recessive	TT/CC + CT					0.836 (0.599‐1.167)	.292
Overdominant	CT/CC + TT					0.964 (0.742‐1.253)	.785
rs2152278				.001	.080		
Codominant	GG	180 (35.7)	238 (47.1)	Reference		1.000	
	GT	250 (49.6)	194 (38.4)	<.001		*1.700 (1.278‐2.261)*	*<.001*
	TT	74 (14.7)	73 (14.5)	.148		1.398 (0.939‐2.080)	.099
Dominant	GT + TT/GG					*1.619 (1.239‐2.114)*	<*.001*
Recessive	TT/GT + GG					1.062 (0.735‐1.535)	.749
Overdominant	GT/TT + GG					*1.556 (1.194‐2.026)*	*.001*
rs8012083				.104	.522		
Codominant	AA	288 (57.1)	311 (61.6)	Reference		1.000	
	AG	185 (36.7)	176 (34.9)	.351		1.127 (0.855‐1.485)	.398
	GG	31 (6.2)	18 (3.6)	.053		1.666 (0.886‐3.133)	.113
Dominant	AG + GG/AA					1.179 (0.904‐1.539)	.224
Recessive	GG/AG + AA					1.593 (0.855‐2.970)	.143
Overdominant	AG + GG + AA					1.085 (0.826‐1.424)	.559
rs7142488				.364	.981		
Codominant	TT	410 (81.3)	415 (82.2)	Reference		1.000	
	TC	93 (18.5)	86 (17.0)	.621		1.157 (0.823‐1.626)	.402
	CC	1 (0.2)	4 (0.8)	.374		0.241 (0.026‐2.209)	.208
Dominant	TC + CC/TT					1.111 (0.794‐1.555)	.539
Recessive	CC/TC + TT					0.235 (0.026‐2.146)	.199
Overdominant	TC/CC + TT					1.166 (0.830‐1.639)	.375

a *P* value of Chi‐square test for comparison of genotype frequency between case and control groups.

b *P* value of Hardy‐Weinberg test in control population.

c *P* values adjusted for age, menarche age, menopausal status, number of pregnancies, number of abortions, history of breastfeeding, and family history of breast cancer in first‐degree relatives in logistic regression analysis.

The italic values represent *P* < .05.

### 3.3 Stratified analysis of SNP genotypes and BC risk

We also examined the effect of SNPs on BC susceptibility in different subgroups of demographic factors (Table 3). CT + TT in rs11622641 was a protective genotype of BC susceptibility in females with age less than or equal to 50 years (OR:0.55, 95%CI:0.33‐0.92), age at menarche less than 14 years (OR:0.55, 95%CI:0.34‐0.88), number of pregnancy more than twice (OR:0.60, 95%CI:0.36‐0.98), and number of abortion less than twice (OR:0.64, 95%CI:0.43‐0.94). BC risk increased in individuals carrying rs7157819 CT + TT and having had two or more abortions (OR:4.19; 95%CI:1.14‐9.49). Subjects at greater risk, who had a younger menopause age (≤50 years), carried variant genotypes TG + GG of rs12880540 (OR:1.80, 95%CI:1.06‐3.07). For CT + TT of rs4144657, the risk of BC was decreased in population who had no menopause (OR:0.65, 95%CI:0.44‐0.95) or without a family history of BC (OR:0.23, 95%CI:0.06‐0.87). The genotype GT + GG of rs2152278 increased the risk of BC in population who was less than 50 years (OR:1.63, 95%CI:1.16‐2.29), age at menarche less than 14 years (OR:1.52, 95%CI:1.10‐2.11), no menopause (OR:1.67, 95%CI:1.18‐2.36).

**Table 3:** Stratified analysis of nine SNPs in *LINC00520* and genetic susceptibility to breast cancer

	rs11622641 (CT + TT/CC)		rs7157819 (CT + TT/CC)		rs12880540 (TG + GG/TT)		rs2152275 (TA + AA/TT)	
	OR (95%CI)^a^	*P* a	OR (95%CI)^a^	*P* a	OR (95%CI)^a^	*P* a	OR (95%CI)^a^	*P* a
Age (year)								
≤50	*0.549 (0.327‐0.923)*	*.024*	1.084 (0.731‐1.606)	.689	1.029 (0.738‐1.433)	.868	0.944 (0.678‐1.316)	.735
>50	0.743 (0.419‐1.315)	.307	1.155 (0.690‐1.934)	.583	1.215 (0.770‐1.915)	.403	1.174 (0.754‐1.827)	.478
Age at menarche (year)								
≤14	*0.549 (0.342‐0.880)*	*.013*	1.137 (0.778‐1.661)	.506	1.173 (0.848‐1.622)	.334	0.891 (0.646‐1.229)	.482
>14	0.976 (0.503‐1.892)	.943	0.902 (0.525‐1.549)	.708	1.024 (0.640‐1.639)	.920	1.488 (0.931‐2.380)	.097
Age at menopause (year)								
≤50	0.764 (0.378‐1.541)	.452	1.149 (0.640‐2.063)	.642	1.800 (1.055‐3.070)	*.031*	1.148 (0.693‐1.900)	.592
>50	0.461 (0.171‐1.248)	.127	0.892 (0.352‐2.258)	.809	1.104 (0.489‐2.492)	.811	1.501 (0.655‐3.439)	.337
Menopause state								
Premenopause	0.604 (0.354‐1.032)	.065	1.142 (0.757‐1.722)	.527	0.932 (0.661‐1.313)	.686	0.902 (0.640‐1.272)	.557
Postmenopause	0.696 (0.401‐1.206)	.196	1.023 (0.634‐1.650)	.927	1.449 (0.941‐2.231)	.092	1.249 (0.823‐1.897)	.296
Number of pregnancies								
≤2	0.785 (0.441‐1.397)	.411	0.941 (0.565‐1.568)	.816	1.289 (0.855‐1.942)	.225	1.195 (0.786‐1.818)	.405
>2	*0.596 (0.363‐0.980)*	*.041*	1.130 (0.755‐1.691)	.551	0.952 (0.670‐1.352)	.783	0.963 (0.677‐1.370)	.836
Number of abortions								
≤2	*0.637 (0.434‐0.935)*	*.021*	0.925 (0.668‐1.282)	.641	1.061 (0.808‐1.393)	.672	1.126 (0.851‐1.490)	.407
>2	0.777 (0.173‐3.495)	.742	*4.192 (1.135‐9.486)*	*.032*	1.609 (0.672‐3.852)	.286	0.591 (0.250‐1.396)	.231
Breastfeeding								
No	0.518 (0.211‐1.270)	.151	1.321 (0.690‐2.527)	.401	1.357 (0.742‐2.484)	.322	1.008 (0.564‐1.803)	.979
Yes	0.719 (0.469‐1.100)	.128	1.022 (0.718‐1.456)	.902	1.078 (0.797‐1.457)	.627	1.027 (0.764‐1.380)	.860
Family history of breast cancer								
No	0.705 (0.472‐1.052)	.087	1.015 (0737‐1.398)	.927	1.050 (0.796‐1.384)	.732	1.007 (0.767‐1.321)	.961
Yes	0.370 (0.095‐1.440)	.152	1.016 (0.272‐3.793)	.981	1.967 (0.673‐5.753)	.216	1.146 (0.388‐3.386)	.805

	rs8008130 (CA + AA/CC)		rs4144657 (CT + TT/CC)		rs2152278 (GT + TT/GG)		rs8012083 (AG + GG/AA)		rs7142488 (CT + TT/CC)	
	OR (95%CI)^a^	*P* a	OR (95%CI)^a^	*P* a	OR (95%CI)^a^	*P* a	OR (95%CI)^a^	*P* a	OR (95%CI)^a^	*P* a
Age (year)										
≤50	1.070 (0.680‐1.684)	.771	0.709 (0.491‐1.024)	.067	*1.630 (1.162‐2.285)*	*.005*	1.212 (0.866‐1.695)	.262	1.248 (0.826‐1.885)	.293
>50	1.466 (0.823‐2.612)	.194	1.284 (0.768‐2.145)	.341	1.471 (0.935‐2.315)	.095	1.218 (0.776‐1.909)	.391	0.878 (0.479‐1.610)	.673
Age at menarche (year)										
≤14	1.462 (0.944‐2.264)	.089	0.829 (0.581‐1.184)	.303	*1.520 (1.096‐2.108)*	*.012*	1.260 (0.912‐1.741)	.161	1.104 (0.732‐1.665)	.637
>14	0.872 (0.473‐1.605)	.660	0.832 (0.483‐1.432)	.506	*1.909 (1.185‐3.074)*	*.008*	1.001 (0.616‐1.627)	.997	0.904 (0.492‐1.659)	.744
Age at menopause (year)										
≤50	1.314 (0.690‐2.506)	.406	1.156 (0.640‐2.090)	.631	1.385 (0.820‐2.338)	.224	0.928 (0.555‐1.551)	.775	0.765 (0.374‐1.565)	.463
>50	1.767 (0.571‐5.472)	.324	2.382 (0.915‐6.201)	.076	1.243 (0.550‐2.812)	.601	1.092 (0.493‐2.418)	.828	0.794 (0.264‐2.387)	.682
Menopause state										
Premenopause	1.085 (0.677‐1.738)	.736	*0.650 (0.444‐0.951)*	*.027*	*1.669 (1.179‐2.363)*	*.004*	1.330 (0.938‐1.887)	.110	1.355 (0.888‐2.067)	.159
Postmenopause	1.424 (0.828‐2.448)	.201	1.415 (0.866‐2.312)	.165	1.452 (0.943‐2.234)	.090	0.988 (0.647‐1.507)	.954	0.773 (0.432‐1.382)	.385
Number of pregnancies										
≤2	0.867 (0.487‐1.545)	.629	1.028 (0.654‐1.614)	.906	*1.707 (1.129‐2.581)*	*.011*	1.332 (0.886‐2.002)	.168	1.131 (0.689‐1.856)	.625
>2	1.494 (0.940‐2.375)	.090	0.745 (0.503‐1.104)	.142	*1.608 (1.130‐2.288)*	*.008*	1.107 (0.780‐1.572)	.569	1.126 (0.707‐1.796)	.617
Number of abortions										
≤2	1.105 (0.766‐1.594)	.592	0.850 (0.626‐1.154)	.296	*1.579 (1.198‐2.080)*	*.001*	1.243 (0.945‐1.636)	.120	1.098 (0.772‐1.562)	.603
>2	1.616 (0.509‐5.135)	.415	0.617 (0.243‐1.571)	.311	*3.249 (1.289‐8.188)*	*.012*	0.958 (0.397‐2.315)	.924	1.055 (0.368‐3.022)	.921
Breastfeeding										
No	0.598 (0.262‐1.367)	.223	0.674 (0.354‐1.285)	.229	*2.180 (1.178‐4.035)*	*.013*	1.344 (0.744‐2.426)	.327	1.052 (0.492‐2.251)	.896
Yes	1.421 (0.957‐2.108)	.084	0.891 (0.638‐1.245)	.500	*1.503 (1.115‐2.027)*	*.008*	1.150 (0.852‐1.554)	.360	1.090 (0.747‐1.590)	.656
Family history of breast cancer										
No	1.161 (0.806‐1.672)	.422	0.896 (0.659‐1.218)	.483	*1.643 (1.244‐2.170)*	<*.001*	1.135 (0.861‐1.498)	.369	1.080 (0.763‐1.528)	.665
Yes	2.196 (0.400‐12.052)	.365	*0.230 (0.061‐0.871)*	*.030*	1.158 (0.399‐3.363)	.788	3.074 (0.967‐9.771)	.057	2.835 (0.487‐16.498)	.246

a *P* values adjusted for age, menarche age, menopausal status, number of pregnancies, number of abortions, history of breastfeeding, and family history of breast cancer in first‐degree relatives in logistic regression analysis.

The italic values represent *P* < .05.

### 3.4 Receptor status and the genotypes of nine SNPs

As shown in Table 4, only the genotype TG (OR:1.62, 95%CI:1.04‐2.52) in rs12880540, the genotype AA (OR:2.74, 95%CI:1.01‐7.42) in rs2152275, and GG (OR:0.31, 95%CI:0.14‐0.69) in rs8012083 were associated with HER‐2 status. There was no discovery between SNPs and ER and PR.

**Table 4:** Association analysis between three SNPs in *LINC00520* and ER, PR, and Her‐2 receptor status in breast cancer patients

SNPs Genotype	ER		*P* a	OR (95%CI)^a^	PR		*P* a	OR (95%CI)^a^	Her‐2		*P* a	OR (95%CI)^a^
	Negative (n = 151)	Positive (n = 341)			Negative (n = 192)	Positive (n = 297)			Negative (n = 138)	Positive (n = 333)		
rs11622641												
CC	135	299		1	171	260		1.000	120	295		1
CT	16	40	.912	1.036 (0.553‐1.939)	21	35	.789	1.083 (0.603‐1.948)	18	36	.572	0.832 (0.439‐1.576)
TT	0	2	1.000	0	0	2	1.000	NC	0	2	1.000	0
CT + TT	16	42	.741	1.110 (0.597‐2.067)	21	37	.619	1.159 (0.649‐2.069)	18	38	.724	0.892 (0.475‐1.678)
rs7157819												
CC	116	252		1	141	225		1	108	249		1
CT	32	82	.604	1.133 (0.706‐1.819)	48	65	.344	0.809 (0.521‐1.255)	26	81	.333	1.285 (0.773‐2.134)
TT	2	7	.987	0.988 (0.248‐3.943)	3	7	.557	0.515 (0.379‐6.052)	4	3	.099	0.273 (0.059‐1.275)
CT + TT	35	89	.626	1.121 (0.709‐1.772)	51	72	.454	0.849 (0.554‐1.301)	30	84	.578	1.148 (0.706‐4.866)
rs12880540												
TT	63	140		1	78	123		1	68	132		1
TG	65	151	.679	1.094 (0.715‐1.673)	81	134	.674	1.090 (0.729‐1.632)	52	154	*.034*	*1.617 (1.037‐2.522)*
GG	23	50	.689	0.887 (0.492‐1.598)	32	40	.324	0.756 (0.434‐1.318)	18	47	.556	1.210 (0.641‐2.283)
TG + GG	88	201	.855	1.038 (0.698‐1.542)	114	174	.972	0.993 (0.682‐1.447)	70	201	.051	1.508 (0.998‐2.278)
rs2152275												
TT	77	186		1	103	158		1	79	172		1
TA	66	129	.267	0.795 (0.530‐1.192)	75	119	.869	1.033 (0.701‐0.524)	54	132	.535	1.143 (0.749‐1.743)
AA	8	26	.583	1.267 (0.544‐2.956)	14	20	.805	0.911 (0.435‐1.908)	5	29	*.048*	*2.735 (1.008‐7.418)*
TA + AA	74	155	.403	0.846 (0.573‐1.251)	89	139	.942	1.014 (0.699‐1.470)	59	161	.241	1.277 (0.849‐1.920)
rs8008130												
AA	128	281		1	161	245		1	114	279		1
AG	22	54	.819	1.066 (0.615‐1.850)	29	47	.856	0.953 (0.568‐1.600)	23	48	.506	0.827 (0.473‐1.447)
GG	1	6	.329	2.919 (0.340‐9.021)	2	5	.444	1.930 (0.359‐9.379)	1	6	.331	2.937 (0.335‐5.729)
AG + GG	23	60	.614	1.148 (0.672‐1.960)	31	52	.962	1.012 (0.614‐1.668)	24	54	.737	1.097 (0.638‐1.887)
rs4144657												
CC	45	96		1	58	83		1	43	94		1
CT	78	183	.903	1.029 (0.653‐1.621)	101	157	.703	1.087 (0.707‐1.673)	74	174	.808	1.059 (0.666‐1.686)
TT	28	62	.855	0.947 (0.530‐1.693)	33	57	.653	1.136 (0.651‐1.982)	21	65	.309	1.381 (0.741‐2.573)
CT + TT	106	245	.976	1.007 (0.653‐1.551)	134	214	.650	1.100 (0.729‐1.659)	95	239	.585	1.132 (0.726‐1.764)
rs2152278												
GG	63	114		1	71	105		1	45	127		1
GT	69	174	.125	1.395 (0.912‐2.135)	97	144	.822	1.048 (0.698‐1.574)	75	154	.105	0.689 (0.439‐1.081)
TT	19	53	.176	1.532 (0.826‐2.841)	24	48	.297	1.367 (0.760‐2.458)	18	52	.978	0.991 (0.519‐1.892)
GT + TT	88	227	.085	1.425 (0.952‐2.133)	121	192	.591	1.112 (0.755‐1.639)	93	206	.186	0.748 (0.486‐1.151)
rs8012083												
AA	78	205		1	109	171		1	76	194		1
AG	61	119	.243	0.783 (0.519‐1.181)	69	111	.632	1.101 (0.743‐1.633)	47	127	.639	1.110 (0.717‐1.719)
GG	12	17	.141	0.551 (0.249‐1.218)	14	15	.261	0.639 (0.292‐1.396)	15	12	*.005*	*0.305 (0.135‐0.693)*
AG + GG	73	136	.139	0.744 (0.503‐1.100)	83	126	.925	1.018 (0.700‐1.481)	62	139	.640	0.907 (0.603‐1.364)
rs7142488												
TT	120	280			154	244			110	272		
TC	31	60	.626	0.884 (0.539‐1.451)	38	52	.539	0.861 (0.535‐1.387)	28	60	.843	0.949 (0.568‐1.586)
CC	0	1	1	0	0	1	1	0	0	1	1	0
TC + CC	31	61	.672	0.899 (0.548‐1.410)	38	53	.587	0.877 (0.545‐1.410)	28	61	.899	0.967 (0.580‐1.614)

a *P* values adjusted for age, menarche age, menopausal status, number of pregnancies, number of abortions, history of breastfeeding, and family history of breast cancer in first‐degree relatives in logistic regression analysis.

The italic values represent*P* < .05.

### 3.5 Molecular typing of BC and the genotypes of nine SNPs

Table 5 showed the association between nine SNPs of *LINC00520* and the different molecular typing states of BC. The genotypic GG (OR:3.58, 95%CI:1.32‐9.69) in rs8012083 increased the risk of triple‐negative BC.

**Table 5:** Association analysis between three SNPs in *LINC00520* and molecular typing of breast cancer

SNPs	Luminal				HER‐2				Triple‐negative			
	Yes (n = 344)	No (n = 127)	*P* a	OR (95%CI)^a^	Yes (n = 78)	No (n = 393)	*P* a	OR (95%CI)^a^	Yes (n = 49)	No (n = 422)	*P* a	OR (95%CI)^a^
rs11622641												
CC	302	113		1.000	71	344		1.000	42	373		1.000
CT	40	14	.973	0.989 (0.511‐1.915)	7	47	.532	0.764 (0.328‐1.777)	7	47	.384	1.486 (0.610‐3.623)
TT	2	0	1.000	0	0	2	1.000	0	0	2	1.000	0
CT + TT	42	14	.867	1.058 (0.549‐2.036)	7	49	.450	0.723 (0.312‐1.676)	7	49	.460	1.395 (0.576‐3380)
rs7157819												
CC	260	97		1.000	60	297		1.000	37	320		1.000
CT	78	29	.765	0.927 (0.564‐1.525)	17	90	.953	0.982 (0.540‐1.786)	12	95	.653	1.178 (0.577‐2.407)
TT	6	1	.564	1.878 (0.221‐5.991)	1	6	.924	0.901 (0.104‐7.775)	0	7	1.000	0
CT + TT	84	30	.867	0.959 (0.588‐1.565)	18	96	.939	0.977 (0.544‐1.756)	12	102	.776	1.109 (0.544‐2.262)
rs12880540												
TT	146	54		1.000	33	167		1.000	21	179		1.000
TG	151	55	.877	1.036 (0.662‐1.622)	33	173	.914	0.971 (0.570‐1.656)	22	184	.931	0.972 (0.507‐1.863)
GG	47	18	.614	0.848 (0.447‐1.609)	12	53	.625	1.203 (0.574‐2.521)	6	59	.874	1.083 (0.407‐2.876)
TG + GG	198	73	.955	0.988 (0.649‐1.503)	45	226	.926	1.024 (0.623‐1.684)	28	243	.986	0.994 (0.538‐1.838)
rs2152275												
TT	186	65		1.000	42	209		1.000	23	228		1.000
TA	132	54	.471	0.853 (0.554‐1.314)	31	155	.958	0.986 (0.590‐1.649)	23	163	.257	1.439 (0.767‐2.699)
AA	26	8	.850	1.086 (0.463‐2.549)	5	29	.773	0.861 (0.312‐2.376)	3	31	.890	1.095 (0.304‐3.948)
TA + AA	158	62	.558	0.883 (0.583‐1.338)	36	184	.894	0.967 (0.591‐1.583)	26	194	.292	1.388 (0.755‐2.553)
rs8008130												
AA	284	109		1.000	69	324		1.000	40	353		1.000
AG	54	17	.663	1.144 (0.625‐2.092)	9	62	.360	0.701 (0.328‐1.498)	8	63	.636	1.224 (0.530‐2.824)
GG	6	1	.367	2.694 (0.312‐3.239)	0	7	.999	0	1	6	.884	1.181 (0.126‐2.109)
AG + GG	60	18	.490	1.229 (0.685‐2.204)	9	69	.220	0.625 (0.294‐1.326)	9	69	.626	1.219 (0.550‐2.701)
rs4144657												
CC	96	41		1.000	22	115		1.000	19	118		1.000
CT	186	62	.389	1.234 (0.765‐1.989)	39	209	.934	0.976 (0.546‐1.743)	23	225	.251	0.675 (0.345‐1.321)
TT	62	24	.973	1.011 (0.550‐1.858)	17	69	.420	1.340 (0.657‐2.731)	7	79	.289	0.603 (0.237‐1.535)
CT + TT	248	86	.496	1.170 (0.745‐1.838)	56	278	.826	1.064 (0.614‐1.842)	30	304	.193	0.656 (0.348‐1.237)
rs2152278												
GG	117	55		1.000	33	139		1.000	22	150		1.000
GT	173	56	.134	1.412 (0.900‐2.217)	33	196	.205	0.706 (0.412‐1.210)	23	206	.536	0.817 (0.429‐1.552)
TT	51	16	.199	1.537 (0.798‐2.959)	12	58	.701	0.865 (0.413‐1.812)	4	66	.158	0.445 (0.145‐1.367)
GT + TT	227	72	.094	1.440 (0.940‐2.206)	45	54	.247	0.742 (0.448‐1.229)	27	272	.308	0.725 (0.391‐1.344)
rs8012083												
AA	203	67		1.000	43	227		1.000	24	246		1.000
AG	125	49	.626	0.896 (0.577‐1.391)	31	143	.678	1.115 (0.667‐1.863)	18	156	.817	1.082 (0.557‐2.100)
GG	26	11	.080	0.476 (0.207‐1.094)	4	23	.950	0.965 (0.314‐2.962)	7	20	*.012*	*3.575 (1.318‐9.698)*
AG + GG	141	60	.340	0.816 (0.538‐1.239)	35	166	.719	1.095 (0.667‐1.797)	25	176	.324	1.359 (0.739‐2.498)
rs7142488												
TT	281	101		1.000	63	319		1.000	38	344		1.000
TC	62	26	.749	0.918 (0.542‐1.553)	15	73	.905	1.039 (0.552‐1.956)	11	77	.737	1.135 (0.542‐2.378)
CC	1	0	1	0	0	1	1	0	0	1	1	0
TC + CC	63	26	.793	0.932 (0.551‐1.576)	15	74	.934	1.027 (0.546‐1.932)	11	78	.765	1.120 (0.535‐2.345)

a *P* values adjusted for age, menarche age, menopausal status, number of pregnancies, number of abortions, history of breastfeeding, and family history of breast cancer in first‐degree relatives in logistic regression analysis.

The italic values represent*P* < .05.

### 3.6 Analysis of haplotype

The haplotype analysis was conducted to evaluate the combined effect of four *LINC00520* SNPs. As shown in Table 6, C_rs7157819_T_rs12880540_A_rs2152275_T_rs11622641_ was the common haplotype in cases and controls with a frequency of 0.05% and 0.078%, respectively, and could decrease BC risk by 37% (OR:0.63, 95%CI:0.44‐0.91). However, other results showed that no haplotype was associated with BC risk with *P* < .05.

**Table 6:** Haplotype analysis of four SNPs in *LINC00520*

Gene	Haplotype^a^	Cases (%)	Controls (%)	χ^2^	*P*	OR (95%CI)
*LINC00520*	C G A C	174.34 (0.173)	164.71 (0.163)	0.349	.554	1.073 (0.849‐1.356)
	C G T C	53.43 (0.053)	47.25 (0.047)	0.409	.522	1.140 (0.763‐1.704)
	C T A C	29.54 (0.029)	19.89 (0.020)	1.948	.163	1.502 (0.845‐2.671)
	C T A T	50.10 (0.050)	77.68 (0.077)	6.316	*.012*	*0.627 (0.435‐0.905)*
	C T T C	556.93 (0.553)	572.14 (0.566)	0.418	.518	0.943 (0.790‐1.126)
	T G T C	128.34 (0.127)	112.65 (0.112)	1.193	.275	1.162 (0.887‐1.522)

Abbreviation: SNPs, single‐nucleotide polymorphisms.

a The sequence of SNP locus is rs7157819, rs12880540, rs2152275, and rs11622641.

The italic values represent*P* < .05.

### 3.7 Gene‐reproductive factor interaction analysis

We further explored the interaction between functional SNPs and other factors on BC risk using multifactor dimensionality reduction (MDR) analysis (Table 7). The optimal gene‐environment interactive model with testing balance accuracy (TBA) of 0.70 and cross‐validation consistency (CVC) of 7/10 revealed that the number of pregnancies more than twice with number of abortions more than twice carrying rs2152278 T allele had 5.24 times higher risk of BC over those without above characteristics.

**Table 7:** Interaction analyses between the functional SNPs and environmental factors associated with breast cancer by MDR

Model	TBA	CVC	χ^2^	*P*	OR (95%CI)
Number of pregnancies	0.596	10/10	31.413	*<.001*	*2.049 (1.592‐2.637)*
Number of pregnancies, number of abortions	0.688	10/10	143.543	*<.001*	*4.935 (3.774‐6.454)*
Number of pregnancies, number of abortions, rs2152278	0.696	10/10	154.784	*<.001*	*5.239 (4.006‐6.853)*

Note

The best model was selected as the one with the maximum testing balance accuracy and maximum cross‐validation consistency.

In this study, the best interaction model was the three‐factor model including number of pregnancies‐number of abortions‐*LINC00520* rs2152278.

Abbreviations: CVC, cross‐validation consistency; MDR, multifactor dimensionality reduction; SNPs, single‐nucleotide polymorphisms; TBA, testing balance accuracy.

### 3.8 Functional correlation of rs12880540 genotypes with the relative expression of *LINC00520* in plasma

**Figure 1:**
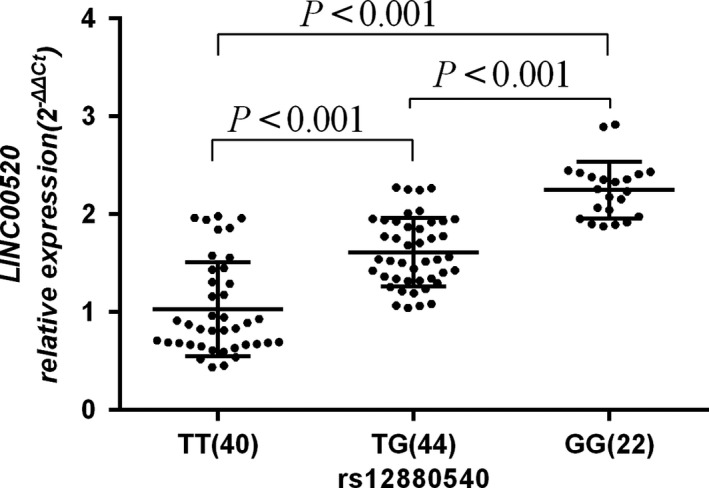
The relative expression of *LINC00520* with different rs12880540 genotypes in plasma. The relative mRNA expression of *LINC00520* levels in blood plasma from 106 cancer‐free controls was significantly higher in the TG (1.61 ± 0.35) and GG genotypes (2.25 ± 0.29) than the TT genotype (1.03 ± 0.48) (all *P* < .001)

As shown by qRT‐PCR analyses (Figure 1), the average relative expression of *LINC00520* in the plasma of individuals with rs12880540 TG (1.61 ± 0.35, n = 44) and rs12880540 GG (2.25 ± 0.29, n = 22) was both higher (both *P* < .0029) than those only with rs12880540 TT (1.03 ± 0.48, n = 40). Furthermore, with the increase of C genotype, *LINC00520* expression appeared to be elevated.

### 3.9 Effect of rs12880540 mutation on the binding of miR‐3122 and ***LINC00520***

**Figure 2:**
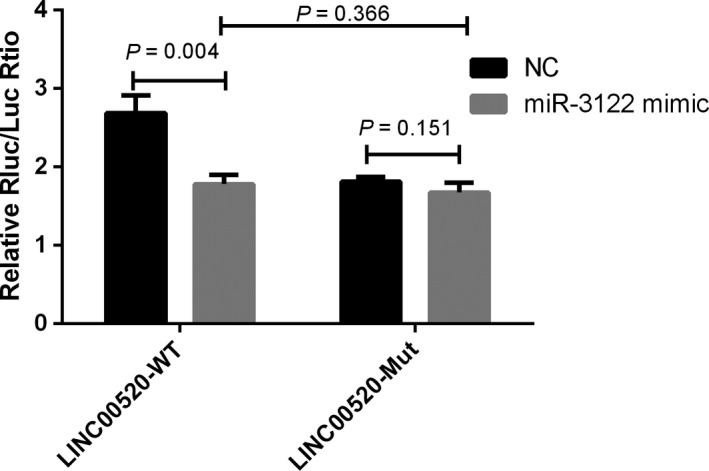
The effect of rs12880540 on the binding of miR‐3122 and *LINC00520* revealed by luciferase reporter assays

As the database predicted, rs12880540 G > T mutation could gain/loss a binding site for miR‐3122 and *LINC00520*, which might regulate the expression of *LINC00520* (Figure 2). To investigate the effect of rs12880540 on the binding of miR‐3122 and *LINC00520*, we constructed luciferase reporter containing part of *LINC00520* covering rs12880540 G > T allele co‐transfected with miRNA mimics in 293T cells, measured and calculated the relatively luciferase activity. Luciferase reporter assays displayed that the luciferase activity of the construct with the risk rs12880540 G allele decreased when transfected with miR‐3122 mimics and there was no change when transfected with NC, which not indicated that rs12880540 G would affect the combination of miR‐3122 and *LINC00520*.

## 4 DISCUSSION

So far as we know, no association between genetic variation of *LINC00520* and BC susceptibility has been explored. In the study, a case‐control research was conducted to analyze the association between *LINC00520* genetic susceptibility and the risk of BC. After adjusted by age, menarche age, menopausal status, number of pregnancies, number of miscarriages, history of breastfeeding, and family history of BC, the study found that the variation of rs11622641 allele was statistically associated with a reduced risk of BC. The variation of rs12880540 and rs2152278 alleles increased the risk of BC. The results showed that TG, rs2152275 genotype TG, and rs8012083 genotype AA of rs12880540 genotype GG were significantly correlated with the positive status of Her‐2 receptor. Haplotype analysis showed that *LINC00520* C_rs7157819_ T_rs12880540_ A_rs2152275_ T_rs11622641_ haplotype could reduce the risk of BC. The homozygous mutant GG at rs8012083 site could significantly increase the risk of triple‐negative BC. A genetic variant of rs8012083 in *LINC00520* could be used as a biomarker for triple‐negative BC. However, the mechanism of its influence on the susceptibility to triple‐negative BC needed to be further studied.

Previous studies have shown that SNP could affect the level of genes expression through allelic changes, thus participating in the carcinogenesis of tumor. Compared with rs619586 A allele, the expression of *MALAT1* in individuals carrying rs619586 G allele was lower, which might be related to the decreased susceptibility to BC.^20^ The G risk gene of rs6983267 site of lncRNA *CCAT2* could increase the expression of *CCAT2*, which was related to the risk of colorectal cancer.^21^ The results showed that the genotype GG at rs12880540 site significantly increased the risk of BC compared with the genotype TT. The relative expression of *LINC00520* showed a linear trend with the increase in the number of rs12880540 mutations. It was suggested that *LINC00520* gene polymorphism could increase the risk of BC and affect the expression of *LINC00520* in plasma, which played an important role in the occurrence and development of BC, consistent with the results of the increased expression of *LINC00520* in BC.^19^

The SNP mutations could change the activity of lncRNAs transcriptional regulatory region and affect the expression of lncRNAs by changing the secondary structure of lncRNAs to obtain or lose the binding site of miRNAs, thus affecting the development, metastasis, and prognosis of tumor.^22^, ^23^, ^24^ The biological function of the secondary structure and the miRNA binding ability of the SNPs of the *LINC00520* were predicted using the lncRNASNP2 database (http://bioinfo.life.hust.edu.cn/lncRNASNP) and the RNAfold website (http://rna.tbi.univie.ac.at/cgi-bin/RNAWebSuite/RNAfold.cgi). The predicted results showed that the secondary structure of the *LINC00520* has changed after the mutation of the rs12880540 allele G to T, while the two binding sites of the miR‐92a‐2‐5p and the miR‐4648 and the four binding sites of the miR‐3122, the miR‐3913‐5p, the miR‐4259, and the miR‐4425 were lost. The results of double luciferase reporter gene experiment showed that *LINC00520* and miR‐3122 interact with each other because of T, but the variation of rs12880540 G > T had little effect on the binding ability of *LINC00520* and miR‐3122. Therefore, the effect of rs12880540 G > T on BC susceptibility may not be due to its change in binding ability of *LINC00520* to miR‐3122.

BC is a disease with extremely complex pathogenesis. It is generally believed that BC is the result of the interaction of many factors, such as personal lifestyle, environment, genetic, and reproductive factors.^25^ So far, studies have found individual or family history of smoking, drinking, obesity, menarche at earlier age, late menopausal, infertile, multiple miscarriages, genetic factors BC or ovarian cancer, genetic mutations, endogenous hormone exposure, and exogenous hormone intake (oral contraceptive and hormone replacement therapy), as well as other environmental and reproductive factors as risk factors for BC, while breastfeeding and physical activity were well‐known protective factors.^26^, ^27^, ^28^, ^29^ The results of this study showed that there was obvious interaction between the number of pregnancies, the number of miscarriages, and rs2152278. However, the mechanism of its interaction needs to be further studied.

For the first time, we found the association between *LINC00520* gene polymorphism and BC susceptibility. The following were advantages of this study. First of all, all the cases included in this study were new cases of BC with definite pathological diagnosis, which was beneficial to the control of current cases‐new cases bias. Secondly, the health control randomly selected from the community chronic disease survey of 20 000 people in Henan Province was used in this study. The age matching (±2 years old) between the case and the control made basic characteristics of the two groups similar, which was helpful to reduce confusion bias. Finally, 5% of the DNA samples were randomly selected for repeated experiments, and the different genotypes of each SNP were randomly selected for sequencing, so as to verify the consistency of the experimental results.

Inevitably, the study also has some limitations. The number of samples was 1009 and this study only aimed at the Chinese Han population, leading to the limited representativeness and universality of this study. Therefore, a larger sample size is needed to verify the experimental results of this study. Secondly, this study only aimed at the Chinese Han population. Due to ethnic or regional differences, the results of this study need to be verified by other regions and ethnic groups. Finally, because of the lack of relevant research of the *LINC00520* and adequate reference and related biological function theory support, the research on the function of the *LINC00520* gene only has a preliminary foundation study, which needs further research.

In conclusion, a genetic variant of rs8012083 in *LINC00520* could be used as a biomarker for triple‐negative BC after further evaluation of diagnostic tests. The genetic variant of *LINC00520* was related to the susceptibility of BC, and rs12880540 might affect the corresponding mRNA expression of lncRNA *LINC00520*.

## CONFLICT OF INTERESTS

None declared.
