# Supplementary figures and images for: Characterization of lncRNA LINC00520 and functional polymorphisms associated with breast cancer susceptibility in Chinese Han population
Source: Cancer Med. 2020 Jan 29;9(6):2252–68. doi: 10.1002/cam4.2893 (PMC7064040; doi:10.1002/cam4.2893)

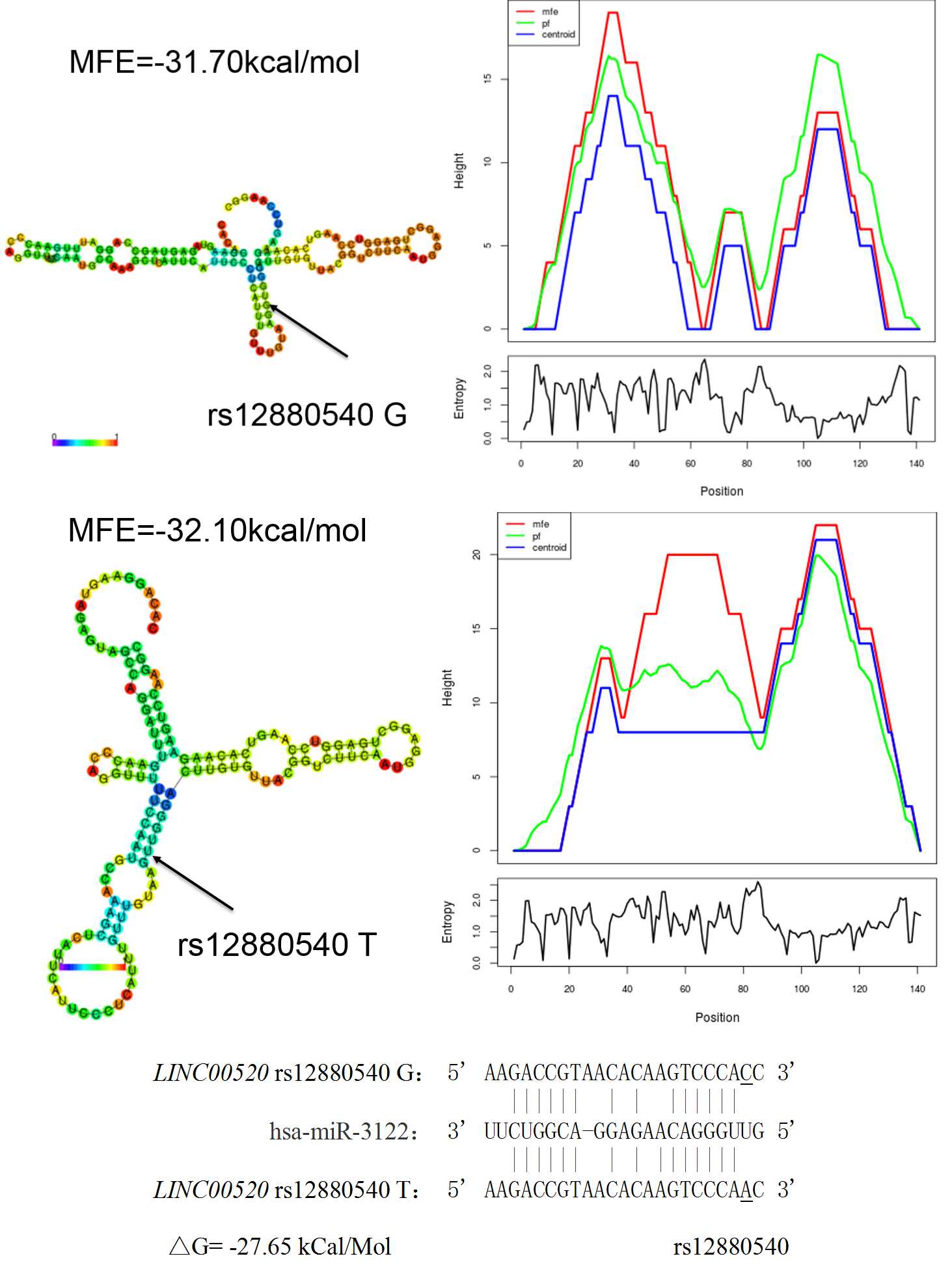

Supplement: Supplementary file 1 [file CAM4-9-2252-s001.jpg]

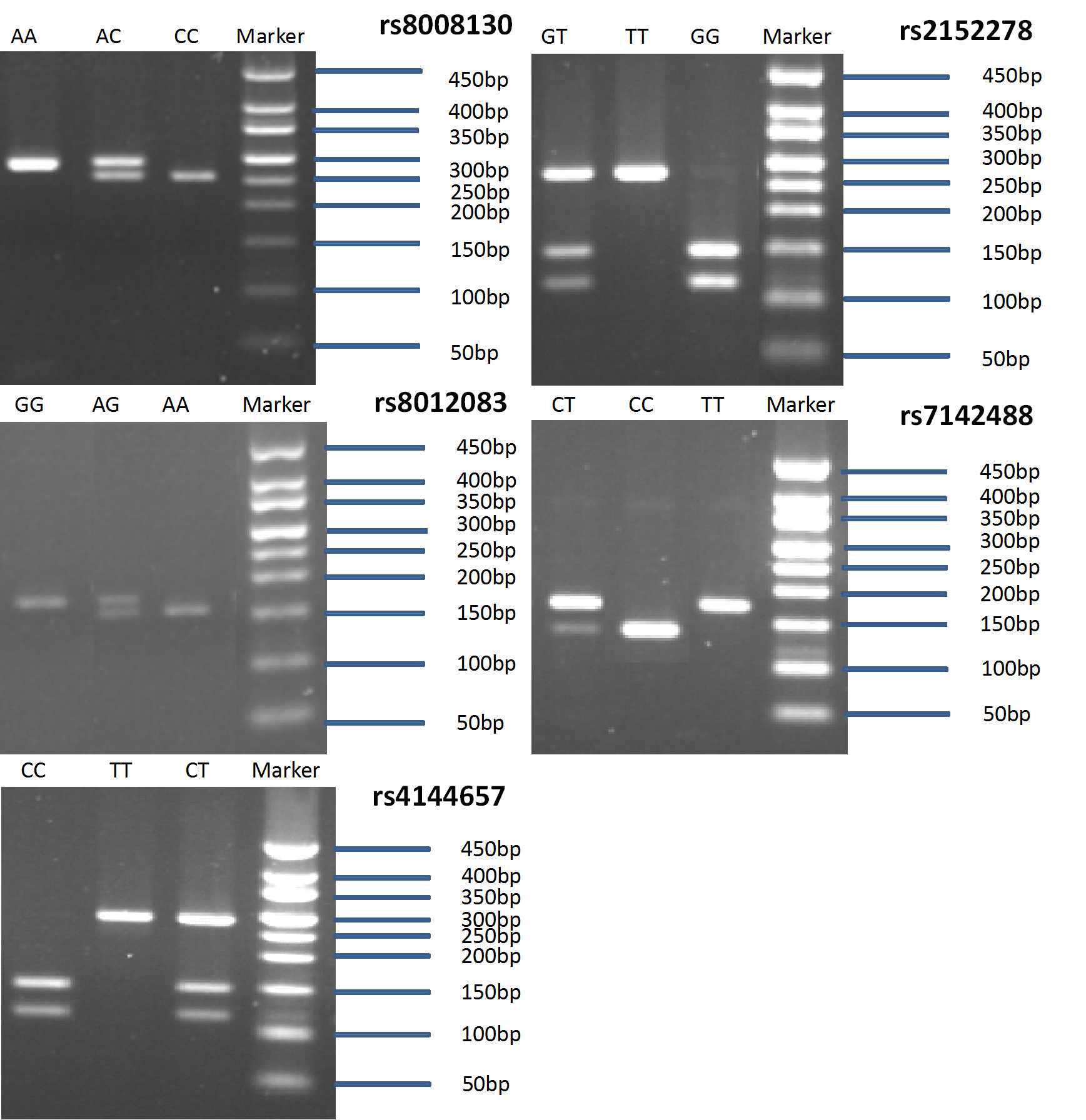

Supplement: Supplementary file 2 [file CAM4-9-2252-s002.jpg]

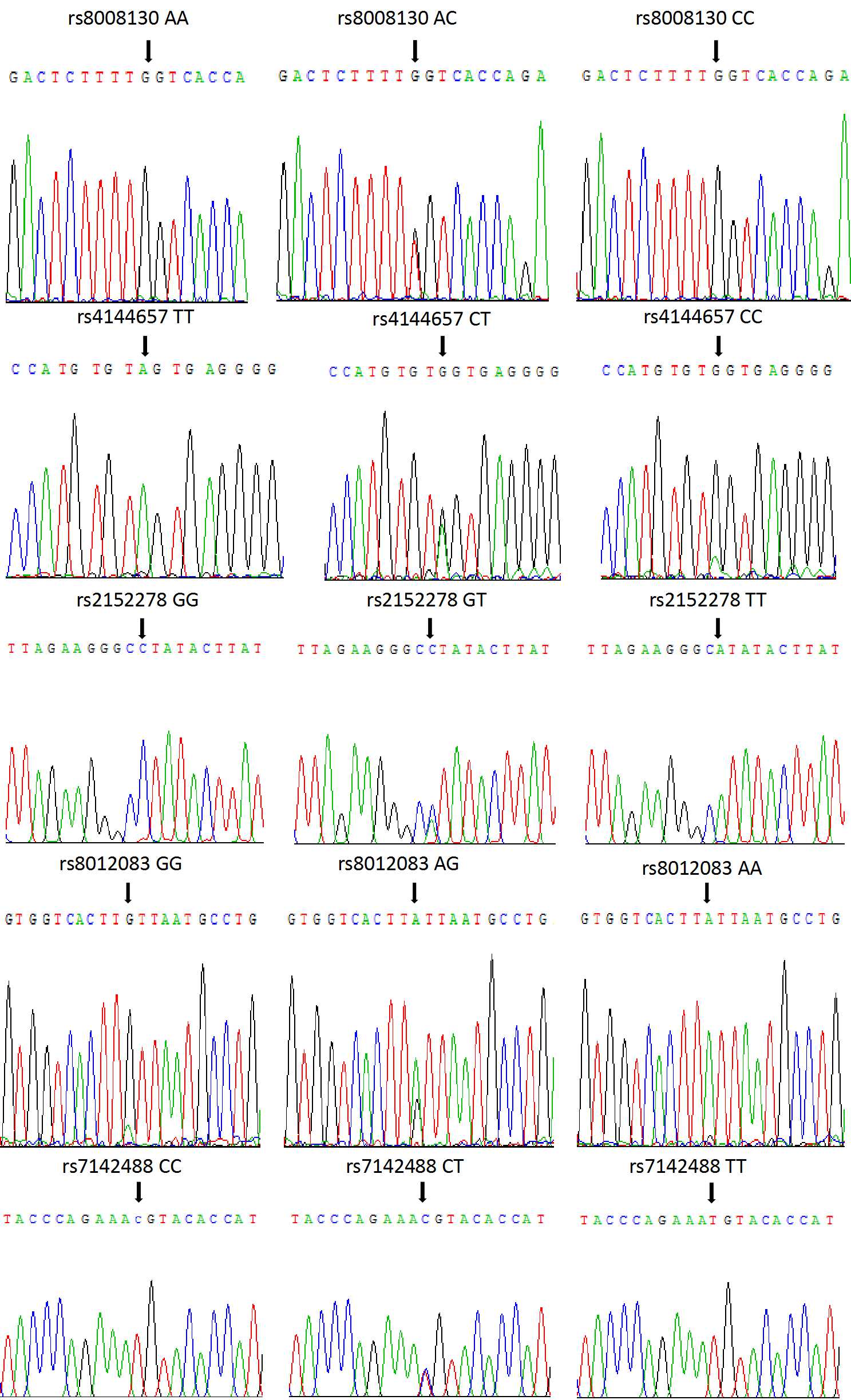

Supplement: Supplementary file 3 [file CAM4-9-2252-s003.jpg]
