# Supplementary material for: Characterization of lncRNA LINC00520 and functional polymorphisms associated with breast cancer susceptibility in Chinese Han population
Source: Cancer Med. 2020 Jan 29;9(6):2252–68. doi: 10.1002/cam4.2893 (PMC7064040; doi:10.1002/cam4.2893)
Supplement: Supplementary file 4 [file CAM4-9-2252-s004.doc]

**S 1.** The SNPs in *LINC00520* may affect the biological function of miRNA binding.

| SNP | MAF | △Energy  (kCal/Mol) | miRNA | Energr(kCal/Mol) | Score | Effect |
| --- | --- | --- | --- | --- | --- | --- |
| rs11622641 | 0.10 | 0.6 | [hsa-miR-6842-3p](http://bioinfo.life.hust.edu.cn/lncRNASNP#!/mirna_info?mirna=hsa-miR-6842-3p&snp=1) | -21.48 | 155 | Gain |
|  |  |  | hsa-miR-29a-5p | -16.93 | 143 | Gain |
|  |  |  | hsa-miR-4687-3p | -12.54 | 140 | Gain |
|  |  |  | hsa-miR-6762-3p | -34.18 | 168 | Loss |
|  |  |  | hsa-miR-135b-5p | -20.23 | 142 | Loss |
|  |  |  | hsa-miR-647 | -19.43 | 151 | Loss |
|  |  |  | hsa-miR-135a-5p | -18.15 | 141 | Loss |
|  |  |  | hsa-miR-103a-2-5p | -14.63 | 147 | Loss |
| rs7157819 | 0.10 | -2.2 | hsa-miR-5701 | -10.25 | 141 | Loss |
| rs12880540 | 0.34 | 0.4 | [hsa-miR-92a-2-5p](http://bioinfo.life.hust.edu.cn/lncRNASNP#!/mirna_info?mirna=hsa-miR-92a-2-5p&snp=1) | -20.88 | 148 | Gain |
|  |  |  | hsa-miR-4648 | -19.61 | 145 | Gain |
|  |  |  | hsa-miR-3122 | -27.65 | 159 | Loss |
|  |  |  | hsa-miR-3913-5p | -17.9 | 156 | Loss |
|  |  |  | hsa-miR-4259 | -16.84 | 153 | Loss |
|  |  |  | hsa-miR-4425 | -15.17 | 146 | Loss |
| rs7142488 | 0.10 | -0.1 | hsa-miR-6730-5p | -21.24 | 145 | Gain |
|  |  |  | hsa-miR-548m | -11.6 | 147 | Gain |
|  |  |  | hsa-miR-5680 | -14.69 | 154 | Loss |

MAF:minor allele frequency.
