# Supplementary material for: Characterization of lncRNA LINC00520 and functional polymorphisms associated with breast cancer susceptibility in Chinese Han population
Source: Cancer Med. 2020 Jan 29;9(6):2252–68. doi: 10.1002/cam4.2893 (PMC7064040; doi:10.1002/cam4.2893)
Supplement: Supplementary file 5 [file CAM4-9-2252-s005.doc]

**S 2.** The basic information of SNPs at *LINC00520.*

| SNP ID | Ancestral Allele | position | NCBI | MAF（CHB） | Genotype Method |
| --- | --- | --- | --- | --- | --- |
| rs11622641 | T/C | 14:55793315 | intron variant | 0.10 | SNPscan |
| rs7157819 | T/C | 14:55780875 | downstream variant 500B | 0.10 | SNPscan |
| rs12880540 | G/T | 14:55781488 | nc transcript variant | 0.34 | SNPscan |
| rs2152275 | A/T | 14:55791248 | intron variant | 0.24 | SNPscan |
| rs8008130 | A/C | 14:55787267 | intron variant | 0.16 | CRS-RFLP |
| rs4144657 | T/C | 14:55781737 | nc transcript variant | 0.30 | PCR-RFLP |
| rs2152278 | T/G | 14:55785344 | intron variant | 0.28 | PCR-RFLP |
| rs8012083 | G/A | 14:55791828 | intron variant | 0.24 | CRS-RFLP |
| rs7142488 | C/T | 14:55781292 | nc transcript variant | 0.10 | PCR-RFLP |

MAF:minor allele frequency, CRS-RFLP: created restriction site PCR, PCR-RFLP: PCR-restriction fragment length polymorphism.
