# Supplementary material for: Characterization of lncRNA LINC00520 and functional polymorphisms associated with breast cancer susceptibility in Chinese Han population
Source: Cancer Med. 2020 Jan 29;9(6):2252–68. doi: 10.1002/cam4.2893 (PMC7064040; doi:10.1002/cam4.2893)
Supplement: Supplementary file 6 [file CAM4-9-2252-s006.doc]

**S 3.** Primer design of five SNPs in *LINC00520* and selection of restriction endonuclease.

| SNP | Genotype Method | annealing temperature(°C) | primer (5′-3′) | restriction enzyme |
| --- | --- | --- | --- | --- |
| rs8008130 | CRS-RFLP | 56.5 | FP: TTTGTAGATGCATCACTCTGGGTAC  RP: AATCTTTGAATCCACCTATGAACC | BstEII |
| rs4144657 | PCR-RFLP | 58.2 | FP: CTCAGCAAACCTCCAGCCAAGTAGT  RP: ACACAGTGACAACAGCCACCAATGT | XcmI |
| rs2152278 | PCR-RFLP | 62.1 | FP: GGTTAAGACGTGGTGCAGGAGGG  RP: ATGAACGCCAGTTTGGGTTTGTCT | HaeIII |
| rs8012083 | CRS-RFLP | 54.0 | FP: TGAGGAGATAAGGAGGGTAT  RP: GGGTTGAGGTCAGGCATT | VspI |
| rs7142488 | PCR-RFLP | 57.0 | FP: AGTTTATTTTGCTCCTTGCTGTTCT  RP: GCCACCTCTGACCACTCGAC | TaiI |

CRS-RFLP: created restriction site PCR, PCR-RFLP: PCR-restriction fragment length polymorphism, FP: Forward primers, RP: Reverse primers.
