# Supplementary material for: Characterization of lncRNA LINC00520 and functional polymorphisms associated with breast cancer susceptibility in Chinese Han population
Source: Cancer Med. 2020 Jan 29;9(6):2252–68. doi: 10.1002/cam4.2893 (PMC7064040; doi:10.1002/cam4.2893)
Supplement: Supplementary file 7 [file CAM4-9-2252-s007.doc]

**Supplementary figure 1.** The centroid secondary structure of *LINCOO520* rs12880540 T/G and biological function prediction results.

**Supplementary figure 2.** The representative images of PCR-RFLP or CRS-RFLP techniques for five SNPs in LINC00520.

**Supplementary figure 3.** The sequencing results and polymorphisms distributions of five SNPs in LINC00520.
